# Post-Mortem Toxicology: A Systematic Review of Death Cases Involving Synthetic Cannabinoid Receptor Agonists

**DOI:** 10.3389/fpsyt.2020.00464

**Published:** 2020-05-25

**Authors:** Arianna Giorgetti, Francesco Paolo Busardò, Roberta Tittarelli, Volker Auwärter, Raffaele Giorgetti

**Affiliations:** ^1^Legal Medicine and Toxicology, University-Hospital of Padova, Padova, Italy; ^2^Institute of Forensic Medicine, Forensic Toxicology, Medical Center, University of Freiburg, Freiburg, Germany; ^3^Section of Legal Medicine, Department of Excellence SBSP, University Politecnica delle Marche of Ancona, Ancona, Italy; ^4^Unit of Forensic Toxicology, SAIMLAL Department, Sapienza University of Rome, Rome, Italy; ^5^Faculty of Medicine, University of Freiburg, Freiburg, Germany

**Keywords:** forensic toxicology, novel psychoactive substances, synthetic cannabinoids, post-mortem examination, toxicological significance score

## Abstract

**Background:**

Synthetic cannabinoid receptor agonists (SCRAs) have become the largest group of new psychoactive substances monitored by the European Union Early Warning System. Despite the wide diffusion on the market, data regarding effects, toxicities, and mechanisms as well as toxic/lethal doses are still scarce.

**Methods:**

A comprehensive literature search for articles published up to January 2019 was performed in multiple electronic databases. Only cases of death in which toxicological analyses revealed the presence of SCRAs in blood or urine and at least an external examination was performed, including those occurred in emergency departments, were included.

**Results:**

Of 380 studies identified, 354 were excluded, while 8 additional manuscripts were included through the screening of relevant references cited in the selected articles. A total number of 34 manuscripts (8 case series and 26 case reports) were included.

**Conclusions:**

Typical toxic ranges for SCRAs have not been so far identified, and the results of toxicological analyses should be interpreted with caution. In death cases involving SCRAs, a thorough post-mortem examination is a prerequisite to assess the role of the substance use in the deceased and to identify a probable mechanism of death. Even after a comprehensive analysis of clinical, circumstantial, toxicological, and autoptic data, the cause and manner of death remain unclear in some cases.

## Introduction

Synthetic cannabinoids or synthetic cannabinoid receptor agonists (SCRAs) are a heterogeneous group of compounds designed to mimic the effects of *delta*-9-tetrahydrocannabinol (Δ9-THC) by binding to the cannabinoid receptors CB_1_ and CB_2_. In contrast to Δ9-THC, a partial agonist at the CB_1_ and CB_2_ receptors, most of the SCRAs marketed so far are full agonists at these receptors and additionally show much higher potency (1, 2). Since their first detection in herbal blends in 2008 (3, 4), they have become the largest group of new psychoactive substances (NPS), with 190 compounds reported to the European Monitoring Centre for Drugs and Drug Addiction (EMCDDA) until the end of 2018 and an extraordinary dynamic market (5), even though there has been a relative reduction of the rate of new compounds per year (5). It was initially claimed that SCRAs could be “safe” alternatives to marijuana, due to similarities of their pharmacological profile to Δ9-THC and other phytocannabinoids, and lots of compounds binding to the cannabinoid receptors have been synthesized and evaluated regarding their binding affinities and activity in animal or cell models since then (6). However, a huge number of in-vitro and in-vivo studies, reports, and alerts have highlighted severe adverse events and enhanced toxicity (2, 7, 8) prompting the United States Drug Enforcement Administration to classify some of these compounds as Schedule I substances. Signs and symptoms of SCRAs consumption include psychomotor agitation, euphoria, anxiety, confusion, and psychosis on the one side, sedation and loss of consciousness on the other (9, 10). Adverse cardiac effects are among the most frequently encountered adverse reactions after SCRA intake. Particularly, both tachycardia (more frequently) and bradycardia have been reported. Gastro-intestinal symptoms with nausea and vomiting are also common. Moreover, rhabdomyolysis, hyperthermia, and hypothermia, seizures, respiratory depression, nephro- and hepatotoxicity were described in combination with SCRA intake (11, 12). Some of these effects might be mediated also by interference with other neurotransmitter pathways, since certain SCRAs can also bind to glutamatergic, serotonin (5-HT), opioid, and both adrenergic and cholinergic receptors and to calcium, sodium, potassium channels (13).

Several cases of intoxication have been reported caused by, e.g., MDMB-CHMICA and AB-CHMINACA, which can cause severe symptoms requiring hospitalization and prolonged recovery time (14). Other compounds, such as Cumyl-PEGACLONE, have been suggested as “relatively safe” due to the low number of poisonings despite the abundant presence in herbal blends (25–30% of tested products) and their widespread use (prevalence of 29% in samples positive for SCRAs, including testing for driving under the influence, insult, and threat, criminal offenses). Moreover, the role of the SCRA was deemed minor or contributory in the majority of death cases (15).

Despite the wide diffusion in the market, data regarding effects, toxicities, and mechanisms as well as toxic/lethal doses are still scarce, making SCRAs one of the most “unpredictable” classes of substances (16). Moreover, only few studies regarding the time of detectability, the diffusion in tissues, and the post-mortem distribution of the drugs can be retrieved in the literature. The limited knowledge regarding the pharmacodynamics and pharmacokinetics of SCRAs contributes to the difficulties of the interpretation of toxicological results. Furthermore, several aspects, such as interactions among SCRAs or in the combination with other drugs, are difficult to assess in cases of SCRA-related deaths.

To our knowledge, there are no previous detailed review papers, which report fatalities caused by the misuse of synthetic cannabinoids providing circumstantial, analytical data, and complete results of post-mortem examination.

The aim of the present study is to offer an overview of thoroughly investigated fatalities involving SCRAs, considering not only analytical results, but also an in-depth analysis on investigative data, analytical methods, and macro and microscopic findings.

## Materials and Methods

### Literature Search and Inclusion/Exclusion Criteria

In February 2019 a literature search for articles published until January 2019 was performed in electronic databases (Pubmed, Scopus), using the following research terms: “synthetic cannabinoids” AND (death OR fatal OR fatalities OR autopsy OR forensic OR post-mortem). Search was done in English language and duplicates were manually deleted. Titles and abstracts were screened and only cases of death, in which toxicological analyses revealed at least one SCRA in blood or urine, and at least an external examination was performed were included. Patients rushed to the emergency department and subsequently died were also included in the selected cases.

Exclusion criteria were: irretrievability of a full-text; off-topic articles (e.g., death cases in which other NPS, but no SCRAs, were detected); *in vitro*/animal model studies; herbal blends analyses; non-fatal cases of intoxication; books/reviews not including unpublished cases of death due to SCRAs; autopsy/external examination not performed.

### Data Extraction

An electronic database with the selected papers was built in Excel ^®^ (Microsoft Office, 2006). For each included manuscript, authors, title, journal, year, and type of publication (e.g., case report, case series), number of death cases and type of involved SCRA were extracted.

A separate database was built with the retrieved papers and, for each death case, the following information was extracted:

- type of victim, referring to age and sex;
- concentrations of SCRA retrieved during toxicological analyses in central and peripheral blood, urine, and tissues;
- other substances detected in blood;
- circumstantial data (and whatever relevant emerged during the death scene investigation), with particular reference to a history of drug abuse and to the availability of herbal blends/paraphernalia at the scene,
- post-mortem gross and microscopic findings;
- cause, manner, and suggested mechanism of death;
- post-mortem interval (PMI)
- role of the SCRA as described by the authors.

### Data Analysis/Interpretation

Only a descriptive statistic was applied. For each death case, two independent observers assigned a Toxicological Significance Score (TSS) to the involved SC, in accordance to the methodology proposed by Elliott et al. (17). When no agreement was achieved, a third person was consulted. This rating was compared with the likely role in death assigned by the authors.

Information related to toxicological analytical methodology (linearity, calibration curve, accuracy, precision, limit of detection/quantification, matrix effect), in accordance with what suggested by Welter-Luedeke and Maurer (18) were also noted and taken into consideration when evaluating the single cases.

## Results

### Literature Review

**Figure 1:**
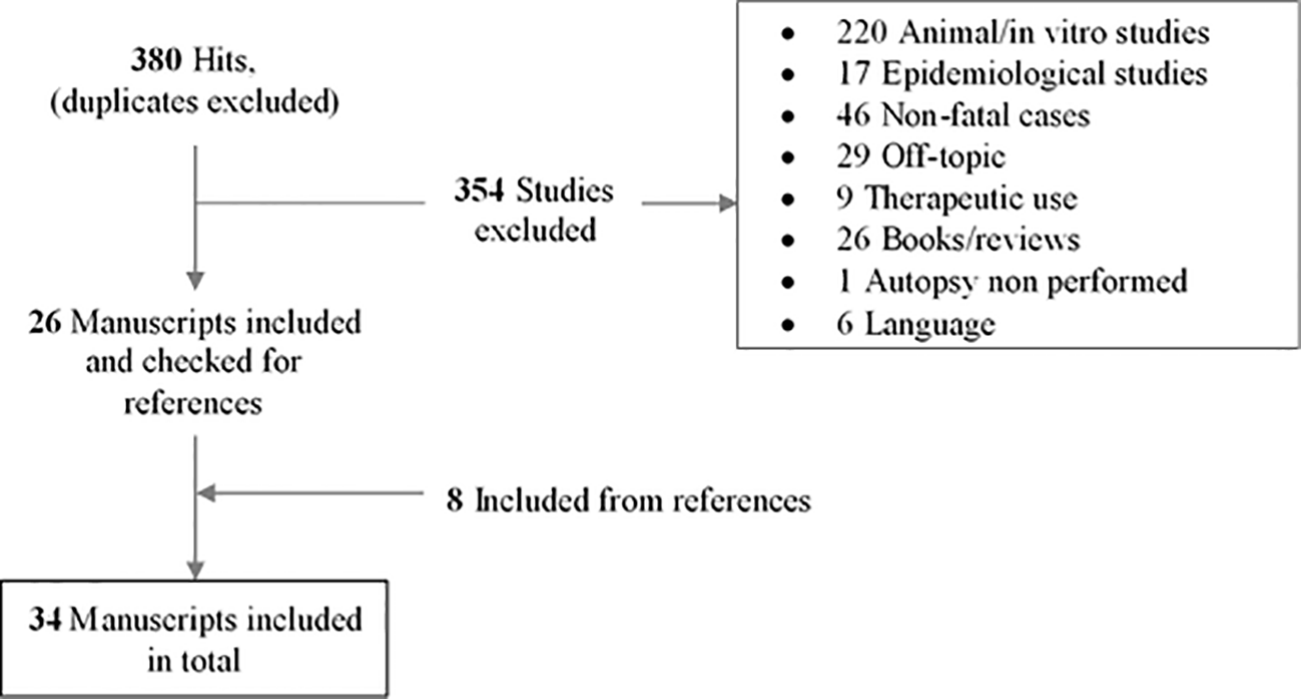
Flow diagram of study identification, screening, and selection.

The literature search resulted in 380 sources after elimination of duplicates. Of these, 354 were excluded applying the criteria listed in “Materials and Methods,” while 8 additional manuscripts were included through the screening of references cited in the selected articles. Details of the literature search are listed in **Figure 1**.

Finally, a total of 34 manuscripts were included (authors, title, journal, date of publication, and involved SCRAs are shown in **Table 1**), corresponding to 74 published cases. Of the 34 manuscripts, 8 consisted in case series and 26 in case reports, including articles only providing new analytical data on previously reported death cases (**Table 1**). **Tables 2** and **3** both refer to the single cases. Particularly, **Table 2** displays the epidemiology of the victim, the involved SCRA(s), other substances detected, anamnestic/circumstantial, and clinical data, macroscopic and microscopic features, cause, and suggested mechanism of death, toxicological significance score, and role of SCRA as suggested by the authors of the paper. In **Table 3**, concentrations in peripheral, central blood, urine, and tissues, together with the PMI are shown.

**Table 1:** Characteristics of the included studies: authors, titles, journal, and year of publication, number of cases reported and type of SCRAs involved (semisystematic names).

Title	Journal	Year	No. of cases	SCRA	Author
A case of intoxication with a mixture of synthetic cannabinoids EAM-2201, AB-PINACA and AB-FUBINACA, and a synthetic cathinone α-PVP.	Leg Med (Tokyo)	2018	–	EAM-2201, AB-PINACA, AB-FUBINACA	Yamagishi et al. (19)
Synthetic cannabinoids: variety is definitely not the spice of life.	J Forensic Leg Med	2018	1	5F-PB-22, 5F-AKB-48	Langford and Bolton (20)
Teens and Spice: A review of adolescent fatalities associated with synthetic cannabinoid use	J Forensic Sci	2018	2	UR-144, XLR-11, JWH-022	Paul et al. (21)
Identification and quantification of predominant metabolites of synthetic cannabinoid MAB-CHMINACA in an authentic human urine specimen.	Drug Test Anal	2018	–	MAB-CHMINACA	Hasegawa et al. (22)
Fatal intoxication by 5F-ADB and diphenidine: Detection, quantification, and investigation of their main metabolic pathways in humans by LC/MS/MS and LC/Q-TOFMS.	Drug Test Anal	2018	1	5F-ADB	Kusano et al. (23)
Post-mortem distribution of the synthetic cannabinoid MDMB-CHMICA and its metabolites in a case of combined drug intoxication.	Int J Legal Med	2018	1	MDMB-CHMICA, EG-018	Gaunitz et al. (24)
Sensitive identification and quantitation of parent forms of six synthetic cannabinoids in urine samples of human cadavers by liquid chromatography–tandem mass spectrometry	Forensic Toxicol	2017	1	5F-ADB, MAB-CHMINACA	Minakata et al. (25)
New challenges in toxicology of new psychoactive substances exemplified by fatal cases after UR-144 and UR-144 with pentedrone administration determined by LC-ESI-MS-MS in blood samples.	Arch Med Sadowej Kryminol	2017	3	UR-144	Rojek et al. (26)
Three fatalities associated with the synthetic cannabinoids 5F-ADB, 5F-PB-22, and AB-CHMINACA	Forensic Sci Int	2017	3	5F-ADB	Angerer et al. (27)
Identification and quantitation of 5-fluoro-ADB, one of the most dangerous synthetic cannabinoids, in the stomach contents and solid tissues of a human cadaver and in some herbal products	Forensic Toxicol	2015	–	5F-ADB, 5F-ADB-PINACA, MAB-CHMINACA	Hasegawa et al. (28)
Postmortem distribution of MAB-CHMINACA in body fluids and solid tissues of a human cadaver	Forensic Toxicol	2015	1	5F-ADB, 5F-ADB-PINACA, MAB-CHMINACA	Hasegawa et al. (29)
Postmortem distribution of AB-CHMINACA, 5-fluoro-AMB and diphenidine in body fluids and solid tissues in a fatal poisoning case: usefulness of the adipose tissue for detection of the drugs in the unchanged forms	Forensic Toxicol	2015	1	AB-CHIMINACA, 5F-AMB	Hasegawa et al. (30)
Fatal poisoning with the synthetic cannabinoid AB-CHMINACA and ethyl alcohol – a case study and literature review	Problems of Forensic Sciences	2016	1	AB-CHMINACA	Gieron et al. (31)
Death after use of the synthetic cannabinoid 5F-AMB	Forensic Sci Int	2016	1	5F-AMB	Shanks and Behonick (32)
Synthetic cannabinoid drug use as a cause or contributory cause of death	Forensic Sci Int	2016	25	JWH-018, AM-2201	Labay et al. (33)
Death associated with the use of the synthetic cannabinoid ADB-FUBINACA.	J Anal Toxicol	2016	1	ADB-FUBINACA	Shanks et al. (34)
Clinical and toxicological findings of acute intoxication with synthetic cannabinoids and cathinones	Acute Med Surg	2016	1	Mepirapim	Fujita et al. (35)
Case report: fatal intoxication with synthetic cannabinoid MDMB-CHMICA	Forensic Sci Int	2016	1	MDMB-CHMICA	Adamowicz (36)
Death due to diabetic ketoacidosis: Induction by the consumption of synthetic cannabinoids?	Forensic Sci Int	2015	1	AB-CHMINACA, AB-FUBINACA, AM-2201, 5F-AMB, 5F-APINACA, EAM-2201, JWH-018, JWH-122, MAM-2201, STS135, THJ2201, UR-144, XLR-11	Hess et al. (37)
High-resolution mass spectrometric determination of the synthetic cannabinoids MAM-2201, AM-2201, AM-2232, and their metabolites in postmortem plasma and urine by LC/Q-TOFMS.	Int J Legal Med	2015	1	MAM-2201, AM-1220, AM-2232	Zaitsu et al. (38)
Case reports of synthetic cannabinoid XLR-11 associated fatalities.	Forensic Sci Int	2015	2	XLR-11	Shanks et al. (34)
Deaths linked to synthetic cannabinoids.	Forensic Sci Med Pathol	2015	3	PB-22	Gerostamoulos et al. (39)
Four postmortem case reports with quantitative detection of the synthetic cannabinoid, 5F-PB-22	J Anal Toxicol	2013	4	5F-PB-22	Behonick et al. (40)
Toxicological findings of synthetic cannabinoids in recreational users.	J Anal Toxicol	2013	1	JWH-210	Kronstrand et al. (16)
K2 toxicity: fatal case of psychiatric complications following AM2201 exposure	J Forensic Sci	2013	1	AM-2201, JWH-018	Patton et al. (41)
An accidental fatal intoxication with methoxetamine	J Anal Toxicol	2013	1	AM-694, AM-2201, JWH-018	Wikström et al. (42)
Detection of JWH-018 and JWH-073 by UPLC-MS-MS in postmortem whole blood casework	J Anal Toxicol	2012	3	JWH-018, JWH-073	Shanks et al. (43)
A fatal case of MAM-2201 poisoning	Forensic Toxicol	2013	1	MAM-2201	Saito et al. (44)
A case of death caused by abuse of a synthetic cannabinoid N-1-naphthalenyl-1-pentyl-1H-indole-3-carboxamide	Forensic Toxicol	2014	1	NNEI	Sasaki et al. (45)
A fatal case involving several synthetic cannabinoids	Toxichem Krimtech	2013	1	JWH-122, JWH-018, JWH-210, MAM-2201, AM-2201, UR-144	Schaefer et al. (46)
A report of novel psychoactive substances in forensic autopsy cases and a review of fatal cases in the literature	Legal Medicine	2017	4	5F-AB-PINACA, 5F-AMB	Kubo S et al. (47)
Sudden cardiac death following use of synthetic cannabinoid MDMB-CHMICA	J Anal Toxicol	2016	1	MDMB-CHMICA	Westin et al. (48)
Analysis and clinical findings of cases positive for the novel synthetic cannabinoid receptor agonist MDMB-CHMICA	Clin Toxicol (Phila)	2016	2	MDMB-CHMICA	Seywright et al. (49)
Identification of 5-Fluoro ADB in human whole blood in four death cases.	J Anal Toxicol	2018	4	5F-ADB	Usui et al. (50)

“-”: further analyses were performed on a previously published case.

**Table 2:** Results of the literature revision for each case.

Age, sex	SCRA(s) in peripheral blood (ng/ml)	Other substances in blood (ng/ml, ethanol g/L)	Anamnestic and Circumstantial data	Clinical data	Gross findings	Histopathology	Cause of death	Suggested mechanism	TSS	Role of SCRA(s) (authors)	Study
-, M	EAM-2201: 0.0566 ± 0.0042; AB-PINACA: 0.0126 ± 0.0001	α-PVP: pos	Dead in a bathtub, with water level lower than the shoulder	No medical history	Lung congestion and edema	NP	Possible intoxication due to synergic effect	NP	TSS U	Possible intoxication due to synergistic effect	Yamagishi et al. (19) .Minakata et al. (25)
35, M	5F-PB-22: pos 5F-AKB-48: pos	Ethanol 3.11	History of schizophrenia, alcohol and alcohol withdrawal, seizures; he was found dead in an alleyway within 30 min of having been given a smoking pipe	Shock advisory defibrillator	Lungs congestion, scattered erosions within the mucosa of the stomach	Confirmation of macroscopic findings	Combination of alcohol and SCRAs	Sudden onset of cardiac arrhythmias or cardiac death	TSS U	Possible contributory role in emotional and mental imbalance	Langford and Bolton (20)
20s, M	5F-ADB: 0.12 (iliac)	–	Found dead in a sitting position in his room. The police found an opened sachet labeled “Heart Shot BLACK” on a table.	No medication history	Unremarkable	NP	Acute circulatory failure after drug inhalation	NP	TSS 2	The SCRA involvement on death is unknown	Usui et al. (50)
50s, M	5F-ADB: 0.23 (iliac)	–	The decedent was lying on the floor in a supine posture. An opened sachet labeled “Heart Shot BLACK” was found on a table.	NP	Ischemic heart disease	NP	Acute circulatory failure after drug inhalation	NP	TSS 2	The SCRA involvement on death is unknown	
20s, M	5F-ADB: 0.16 (iliac)	–	Found dead in a prone position in a hallway, after vomiting and bleeding from nose	No medication or medical history	Unremarkable	NP	Acute circulatory failure after drug inhalation	NP	TSS 2	The SCRA involvement on death is unknown	
50s, M	5F-ADB: 1.38 (iliac)	–	History of schizophrenia under medication, he was found dead in his car in a parking lot while he was holding a plastic pipe and with an unsealed “Heart Shot BLACK” package in his hands	For the treatment of schizophrenia he was taking risperidone, biperiden, and olanzapine.	Unremarkable	NP	Acute circulatory failure after drug inhalation	NP	TSS 2	The SCRA involvement on death is unknown	
14, M	AB-CHMINACA: 8.2 (subclavian)	–	Daily SC intake for 6 months, last use an hour before experiencing sudden cardiac death following an unwitnessed collapse in his bathroom	No history of any other substance use, or any relevant medical, surgical or family history	Dilated cardiomyopathy, cardiomegaly (520 g), bilateral pulmonary edema, bilateral pleural effusion and ascites	Cardiomyocyte hypertrophy, contraction band necrosis, pulmonary edema and pulmonary vascular congestion	SCRA intoxication	Sudden cardiac death and dilated cardiomyopathy	TSS 3	SCRA intoxication	Paul et al. (21)
17, M	UR-144: 12.3 (subclavian), XLR-11: 1.3 (subclavian), JWH-022: 3 (subclavian)	–	Occasional user of SC (“Black Mamba”), last use 4-5 h before death. He was found in his bedroom	NP	Unremarkable	Unremarkable	SCRAs intoxication	Sudden death	TSS 3	NP	
34, M	MAB-CHMINACA: 6.05	Ethanol: 0.3 Quetiapine: pos Nicotine: pos	Severe drugs dependence with multiple admission to mental health hospital; found dead in his room	NP	Massive aspiration of gastric content occluding the airways	NP	Asphyxia due to aspiration of stomach contents into the trachea	Vomiting under reduced consciousness	TSS 3	NP	Hasegawa et al. (22, 28, 29)
53, M	5F-ADB: 0.19 ± 0.04 (*)	Diphenidine: 12 ± 2.6	Found dead at his apartment. Near the deceased were found an open package of a branded herbal blend (“Heart Shot BLACK”).	NP	Unremarkable	NP	Probably SC and diphenidine intoxication	NP	TSS U	Acute mixed intoxication	Kusano et al. (23)
27, M	MDMB-CHMICA: 1.7	Amphetamine: 1050 MDMA: 275 MDA: 22 THC: 9.3 THCCOOH: 65	Eyewitnesses reported that the man fell from the 24th floor of a building.	NP	Multiple injuries to head (including partial debraining), left lung and internal bleeding due to rib fractures.	NP	Fall from height	Psychosis-induced or loss of attention	TSS 1	Under the influence	Gaunitz et al. (24)
30, M	AB-CHMINACA: pos 5F-AMB: pos	Diphenidine 715	Found dead in a parked car	NP	Livor mortis associated with vibices for wide areas of the body surface with a few subcutaneous hemorrhages	NP	NP	NP	TSS U	NP	Minakata et al. (25) .Hasegawa et al. (30)
30s, M	5F-ADB: pos MAB-CHMINACA: pos	–	Found dead at home	NP	Unremarkable	NP	NP	NP	TSS U	NP	Minakata et al. (25)
16, M	UR-144: 2.1	–	He smoked “dope” and a cigarette with UR-144, experienced hallucinations and psychosis, lost control of himself and jumped out of the window from the second floor of the building	NP	Multiple fractures to skull, thoracic and lumbar spine, pubic and ischial bones and multi-organ injuries	NP	Fall from height	Psychosis-induced	TSS 3i	NP	Rojek et al. (26)
22, M	UR-144: 1.4	Pentedrone: 2300	History of alcohol and drugs abuse, previous suicidal ideations; showed mental disorders and aggressive behavior, injured a witness with an axe	NP	NP	NP	Asphyxia due to hanging	Behavioral abnormalities	TSS 1i	NP	
40, M	UR-144: 4	Pentedrone: 290	History of mental instability; psychomotor agitation, aggressive behavior, after smoking a legal high called Orange Flame	Acute toxic liver damage, kidney failure, rhabdomyolysis, disseminated intravascular coagulation, bleeding in the gastrointestinal tract and traumatic hematomas; cardiac arrest	NP	NP	Massive multi-organ failure due to the effect of toxic substances	Behavioral abnormalities with desire of enhancing stimulating effects	TSS 1i	NP	
25, M	5F-PB-22: 0.37	Ethanol: 2.6	History of alcohol and illicit drug use, found dead in his apartment; witnesses reported that the decedent had drunk a lot of alcohol on the evening before his death. Packages of products named “F1,” “Hammer Head,” and “Magic Gold” were found at the scene	NP	Cerebral edema, pulmonary edema, acute blood congestion of internal organs, petechial hemorrhage in eyelids, facial skin and on the lungs	NP	Partial or complete obstruction of the upper airways	Suffocation in presence of SC and ethanol in a state of unconsciousness	TSS 2	Direct contribution	Angerer et al. (27)
28, M	AB-CHMINACA: 4.1	Ethanol: 1.45	Extensive consumer of alcohol and illicit drugs, found dead in his flat. Three packages of the herbal blend “Desert Premium Potpourri 2 g” were located besides the decedent	NP	Cerebral edema and pulmonary edema	NP	Mixed ethanol and SC intoxication with fatal outcome	NP	TSS 2	Direct contribution	
41, M	5F-ADB: 0.38	Ethanol: 0.09 Trimipramine: 170 Olanzapine: 41	Methamphetamine user, found at home	NP	Cerebral edema, pulmonary edema, acute blood congestion of internal organs, petechial hemorrhages in eyelids, facial skin and on the lungs	Myocardial cells death, amorphous material in alveoli as a sign of aspiration of stomach content	Coma and subsequent aspiration of vomit	NP	TSS 3	Direct contribution	
-, -	AB-PINACA: 26	α-PVP: 9900 MDPV: 55	NP	NP	NP	NP	Fall from height	NP	TSS U	NP	Kubo et al. (47)
-, -	5F-AMB: 8	α-PVP: 90 AH-7921: 1100	NP	NP	NP	NP	Intoxication	NP	TSS U	NP	
-, -	Mepirapim: 157	6-APB: 2.7 6-MAPB: <1 α-PHP: 338 DL-4662: 138, Sodium valproate: pos	NP	NP	NP	NP	Intoxication	NP	TSS U	NP	
-, -	5F-AMB: <1	DL-4662 <1, α-PHP 15, metamphetamine 30, amphetamine 10	NP	NP	NP	NP	Intoxication	NP	TSS U	NP	
30s, M	AB-CHMINACA: 1.5 (**)	Ethanol: 1.8	Alcohol addiction. After smoking a pipe with a herbal mixture (“Strongman”), he collapsed and had a slurred speech. An hour later, was found with vomit, weak pulse, shallow breathing. About half an hour later, was declared dead by the emergency doctor	NP	Congestion of internal organs and pulmonary edema	NP	Acute cardiorespiratory failure	SNC depression	TSS 3	Intoxication due to alcohol and SCRA consumption	Gieron and Adamowicz (31)
22, M	MDMB-CHMICA: 1.4 (ante-mortem serum)	Mirtazapine: 5.3 THC: 1.5 Cetirizine: pos	Found lifeless 15 minutes after smoking a brown organic powder	Asystole, declared dead due to brain hypoxia	Anoxic brain damage and pneumonia	NP	Sudden cardiac death	NP	TSS 3	Overdose	Westin et al. (48)
44, M	MDMB-CHMICA: 1 (**)	Amitriptyline: 130	History of poor mental health	NP	NP	NP	Suicidal by hanging	NP	TSS 1	Not attributable to SCRA	Seywright et al. (49)
38, M	MDMB-CHMICA: <1 (**)	Ethanol: 2.37 Acetone: < 100 BHB: 249	History of alcoholism: found dead at home	NP	NP	NP	Complications of chronic alcohol abuse and acute alcohol toxicity	NP	TSS 1	Not attributable to SCRA	
34, M	5F-AMB: 0.3 (subclavian)	–	History of ethanol abuse, found supine on the floor. An opened bag of “Apollo” brand herbal incense was found in his pocket	NP	Unremarkable medical history	NP	SC toxicity	NP	TSS 2	Related (SCRA toxicity)	Shanks and Behonick (51)
41, M	JWH-018: 0.11, AM-2201: 2.5	Phenytoin: 8800	Erratic and aggressive behavior, restrained by the police	NP	Cardiomegaly with four chamber dilation	NP	Complications of excited delirium associated with synthetic marijuana use following police arrest and restraint procedures	Delirium-induced	TSSi 2	NP	Labay et al. (33)
23, M	JWH-210: pos	Fentanyl: pos	Single motor vehicle crash, no significant injuries, restrained by the police	NP	NP	NP	Agitated delirium associated with SCRA use following police arrest and restraint procedures	Delirium-induced	TSS 1	NP	
25, M	AM-2201: 0.21 JWH-018: 0.65 JWH-122: pos JWH-210: pos	Ethanol: 0.15 delta-9-THC: 1.1 THC-COOH: 6.0	Unresponsive after a party, recent binge drinking and jumping from a patio. He was found “frozen” after the jump.	NP	No evidence of head, chest or abdomen injury.	NP	Complications of acute ethanol toxicity, acute SCRA toxicity, possible hypothermia	NP	TSS 2	NP	
42, M	XLR-11: pos	Methamphetamine: 730 Amphetamine: 90	Seizure-like activity after methamphetamine and K2 intake	Asystole at emergency arrival	Hemorrhagic pulmonary edema, obesity, cardiomegaly, moderate coronary artery atherosclerosis, hepatomegaly, splenomegaly, cholesterolosis, abrasions on hip and face	Macrosteatosis, chronic active hepatitis	Mixed drug intoxication	NP	TSS 1	NP	
55, M	AM-2201: 17 JWH-018: 0.47	Chlorpheniramine: < 100, Paroxetine: pos, Benzodiazepines: pos, Alprazolam: <50, Aripiprazole pos	Recent chest pain and heart palpitations in history of cardiac problems	Diagnosed type 2 diabetes	Coronary artery disease, obesity, cholelithiasis, rectal polyps, diabetes	NP	Ischemic heart disease, obesity diabetes SCRA toxicity	NP	TSS 1	NP	
34, M	XLR-11: pos UR-144: pos	Ethanol 0.03 Lidocaine pos	Collapse on public street, after intake of alcohol and drugs	NP	Presence of a needle puncture in the left antecubital fossa	NP	SCRAs and alcohol	NP	TSS U	NP	
21, M	JWH-018: pos	Ethanol: 0.013 Delta-9-THC: 7 THC-COOH: 17 Caffeine: pos Theobromine: pos Atropine: 110	Decedent found unresponsive in bedroom	NP	Pulmonary congestion, vomitus in upper airway, aspiration pneumonia, patchy alveolar hemorrhage	Pneumonitis	Mixed drug intoxication, aspiration pneumonia	NP	TSS 1	NP	
24, M	JWH-122: pos JWH-210: pos AM-2201: 0.16	Delta-9-THC: 2.7 THC-COOH: 6.4 Caffeine: pos Nicotine: pos Cotinine: pos	Learning disability, found lying prone on floor of his bedroom	NP	Bloody froth in airway, cardiomegaly	NP	SCRAs adverse effects	NP	TSS 2	NP	
38, M	UR-144: pos	Amphetamine: pos Alprazolam: pos Citalopram/escitalopram: 130 Hydrocodone: 26 Morphine (free): pos	Found deceased lying on bed, after “partying” with others	NP	NP	NP	Mixed drug intoxication	NP	TSS U	NP	
24, M	JWH-210: pos AM-2201: 1.1	Fluoxetine: 620 Norfluoxetine: 520 Phenobarbital: pos Benzodiazepines: pos Diphenydramine: pos Methadone: pos	Found unresponsive in bed, in therapy with methadone	NP	NP	NP	Mixed drug intoxication	NP	TSS U	NP	
NP, M	XLR-11: pos	Delta-9-THC: 4.3 THC-COOH: 38 Oxycodone 420 Haloperidol 4.7 Fluoxetine 1300 Norfluoxetine 370 Trazodone 250	Death in private residence	NP	Congested lungs, froth in airway, bilateral pleural effusions, remote lesions in brain, variable discoloration of liver	NP	Mixed drug intoxication	NP	TSS 1	NP	
56, F	XLR-11: pos	–	Previously afflicted with cancer, noncompliant diabetic, shortness of breath after smoking “Diablo Spice”	Cardiac arrest at emergency arrival	NP	NP	SCRA abuse, carcinoma of breast, diabetes	NP	TSS U	NP	
15, M	XLR-11: pos	–	Found next to bathtub with the face submerged and vomit from nose	Anoxic brain injury	NP	NP	SC	NP	TSS U	NP	
42, F	AM-2201: 2.8 JWH-018: 0.11	Carbon monoxide: 4300 Caffeine: pos Cotinine: pos	History of alcohol and SC use. Heart disease. Vomit and diarrhea after binge-drinking and smoking “Spice,” then found supine on bedroom floor	NP	Congestion in lungs, fatty liver, cardiomegaly, chronic obstructive pulmonary disease, mild coronary arteriosclerosis	NP	SCRA toxicity	NP	TSS 2	NP	
25, M	JWH-122: pos, JWH-250: 0.23 AM-2201: 7.3	Caffeine: pos Theobromine: pos Nicotine: pos Cotinine: pos	Found prone on bed	NP	Obesity, foam in external nares and pulmonary edema	NP	Adverse effects of drugs	NP	TSS 2	NP	
17, M	JWH-122: pos	THC-COOH: 5.2	Cardio-pulmonary arrest after use of “Legal Phunk”	NP	NP	NP	Sudden death associated with SCRA use	NP	TSS U	NP	
25, M	JWH-122: pos JWH-210: pos AM-2201: 0.22	–	Found unresponsive in the bathroom after vomiting	Anoxic brain injury	NP	NP	Anoxic brain injury due to SCRA toxicity	NP	TSS U	NP	
55, M	AM-2201: 0.13	Hydrocodone < 20	Found unresponsive and cold on garage floor with a large “goose egg” on the back of his head and a small amount of blood	NP	Hypertensive heart disease, blunt force injuries of the head, pulmonary emphysema, obesity, hemangioma in liver	NP	Hypertensive heart disease, blunt force injuries to the head, SCRA presence	NP	TSS 1	NP	
29, M	JWH-122: pos	–	Sweating and vomiting for several days, found dead at home	NP	Coronary artery disease, multiple sharp force injuries to foot	NP	Acute myocardial ischemia, coronary artery disease	NP	TSS 1	NP	
61, F	XLR-11: pos	Ethanol: 0,03 Metoprolol: pos Metoclopramide: trace	Found unresponsive in bed	NP	No autopsy	NP	Atherosclerotic cardiovascular disease	NP	TSS 1	NP	
52, M	JWH-018: 0.28	Chlordiazepoxide: 2000 Nordiazepam: 750 Norchlordiazepoxide: detected Demoxepam: detected Oxazepam: trace detected	Struck by two vehicles while he was crossing a road	NP	No autopsy	NP	Multiple blunt force injuries	NP		NP	
15, F	XLR-11: pos	–	Passenger in auto collision	NP	NP	NP	Multiple injuries	NP	TSS 1	NP	
30, F	XLR-11: pos	Lorazepam: 28 Cotinine: pos Lidocaine: pos	Chest pain	Pulseless ventricular fibrillation, successful resuscitation, acute ST-elevation, myocardial infarction, ventricular fibrillation	Complete occlusion of left anterior descending coronary artery with a thrombus	NP	Acute myocardial infarction due to coronary artery thrombosis	NP	TSS 1	NP	
31, F	JWH-175: 105	MDEA: 217 MDA: 111	Immunosuppression due to kidney transplant, diabetes, consumed a pot brownie, experiencing vomiting and drowsiness, bewilderment. Fell from a fire escape	NP	Subdural hematoma, pelvic fracture, liver laceration, facial and elbow fractures	NP	Multiple blunt traumatic injuries, acute mixed drug intoxication	NP	TSS 1i	NP	
58, M	JWH-210: pos	–	Collapse in a parking after a wavering gait, after smoking K2	Seizure	Atherosclerotic cardiovascular disease and cardiac hypertrophy	NP	Acute myocardial infarction due to coronary artery thrombosis	NP	TSS 1	NP	
41, F	ADB-FUBINACA: 7.3 (*)	THC: 1.1 THC-COOH: 4.7	Aggressive after smoking SC known as “Mojo,” physically restrained by her children, then became unresponsive.	NP	Pulmonary edema, vascular congestion, thrombotic occlusion of the lumen of the left anterior descending coronary artery by hemorrhagic disruption of coronary arterial plaque, ischemia of the anterior left ventricular myocardium	NP	Coronary arterial thrombosis in combination with SCRA use	Block of the artery's blood flow leading to dysrhythmia	TSS 1	Contribution proved by temporal relationship	Shanks et al. (51)
23, M	Mepirapim: 950 (serum)	A-EAPP 3100	Fell asleep after ingestion of the drugs in the restroom; he was found without respiratory signs and was transferred to a hospital	Cardio-pulmonary arrest and confirmed dead approximately 3.5 h after drug use	Congestion of the organs (particularly the lungs) and gastrointestinal bleeding from the stomach into the duodenum	NP	Acute circulatory failure due to SCRA intoxication	Acute circulatory failure	TSS 2	NP	Fujita et al. (35)
25, M	MDMB-CHMICA: 5.6 (ante-mortem blood), MDMB-CHMICA: < 0.2 (0.09 estimated) (post-mortem blood)	Ethanol: 1.48 (ante-mortem blood) Ethanol: 0.81 (post-mortem blood)	History of alcohol and NPSs abuse. After smoking two different SCRAs in a day and drinking a beer, he was wheezing, vomited and then lost consciousness. He was found lying on the floor, without a circulation and a pulse	On hospital admission, he was deeply unconsciousness, limp, circulatory and respiratory inefficient, without deep tendon, pharyngeal and tracheal reflexes. During hospitalization, severe redness of the skin, pathological muscle contraction of chest were observed. Purulent and watery content was expelled and diarrhea and bleeding diathesis	Respiratory, circulatory, heart, kidney and liver failures as well as hypoxic-ischemic damage of the CNS	NP	SCRA intoxication in combination with ethanol	Asystole, loss of consciousness, multiple organ failure and then cardiac arrest	TSS 2	SCRA main cause of poisoning	Adamowicz, (36)
25, M	AB-CHIMINACA: 2.8 AB-FUBINACA: 0.97 AM-2201: <0.1 5F-AMB: 0.19 5F-APINACA: 0.51 EAM-2201: <0.1 MAM-2201: <0.1 STS 135: 0.16 THJ 2201: 0.16	XLR-11 m and UR-144 m in urine	History of SCRA use with previous intoxications, insulin dependent diabetes. Found dead in his apartment	NP	Brain edema, pulmonary edema, subepicardial petechial hemorrhages, hepatic steatosis, aorta angusta, small myocardial scars	Pulmonary congestion and edema, microvesicular steatosis of the liver, Armanni-Ebstein cells in kidneys	Diabetic ketoacidosis, probably following SCRA consumption	Skipping of insulin doses due to intoxicated state or SCRAs induced hyperglicemia	TSS 1	Contributory	Hess et al. (37)
20, M	MAM-2201: 16.3, AM-1220: 140, AM-2232: 0.86	–	10 min after smoking an herbal product, violent behavior. Found dead after 1.5 h	NP	Lungs, heart and liver congestion	NP	NP	NP	Indirect	Unrated contribution	Zaitsu et al. (38)
29, F	XLR-11: 1.4	Diphenhydramine: 81	Known user of SC, found dead on the floor of the bedroom	NP	Unremarkable		SCRA intoxication	NP	TSS 2	Causative	Shanks et al. (34)
32, F	XLR-11: 0.6	–	History of drug abuse (methamphetamine, heroin and SCRAs). The day before, presented to the emergency room with chest pain, nausea, and agitation, diagnosed with anxiety. Later found unresponsive at a friend's house.	NP	Pulmonary edema and congestion, acute visceral congestion and mild pulmonary anthracosis	NP	NP	NP	TSS 2	Probable contributory	
15–35	PB-22: pos	–	Found at home	NP	Unremarkable	Unremarkable	Unascertained	NP	TSS U	Unknown, no competitive cause	Gerostamoulos et al. (39)
15–35	PB-22: pos	–	Found at home	NP	Unremarkable	Unremarkable	Unascertained	NP	TSS U	Unknown, no competitive cause	
15–35	PB-22: pos	–	Found at home	NP	Unremarkable	Unremarkable	Unascertained	NP	TSS U	Unknown, no competitive cause	
20s M	NNEI: 0.99, 0.64 (*)	–	Found dead on the floor of his room, a package containing dried herbal blend labeled “Fairy evolution” was found in the room, previous history of weight loss	No medical history	Marked lungs congestion	Organs and lungs congestion. Lungs: marked congestion and edema, alveolar macrophage infiltrations. Liver: slight lymphocytic infiltrations in the Glisson's sheat. Spleen: arteriolar hyalinizations and severe congestion. Brain: Corpora amylacea. Heart: arteriolar wall hypertrophy, slight interstitial fibrosis and contraction bands	Acute circulatory disturbance induced by NNEI	Hypertension and hyperactivity of cardiac function	TSS 2	SCRA poisoning	Sasaki et al. (45)
17, M	5F-PB-22: 1.1	Ethanol: 0.033, Amiodarone Caffeine (not quantified)	His friends reported that he began gasping for air and then fell to the ground, after SCRA consumption and ethanol intake	NP	Unremarkable		SCRA intoxication	Possible anaphylactic etiology characterized by sudden onset cardiac dysrhythmias or seizure	TSS 2	NP	Behonick et al. (40)
27, M	5F-PB-22: 1.3 (ante-mortem serum)	THC-COOH	History of marijuana use of several times per week.	Acute liver injury, severe coagulopathy, acute kidney injury, acute respiratory failure, hypoxemia, severe anion gap metabolic and lactic acidosis. Brief episode of cardiac arrest, and pulseless electrical activity and poor oxygenation secondary to acute respiratory distress syndrome likely the result of aspiration and pulmonary contusions following chest compressions.	NP	NP	Fulminant liver failure in the setting of THC and SC exposure	Acute hepatic failure caused by the accumulation of a toxic metabolic intermediate still unidentified.	TSS 2	NP	
18, M	5F-PB-22: 1.5 (iliac)	–	Found dead at home after a night of partying, with alcohol intake and SCRAs smoking (K2/Spice)	NP	Bilateral pulmonary vasocongestion and congestion in the abdominal organs (liver, spleen and kidneys)	NP	Sudden death, in association with synthetic cannabinoid use	NP	TSS 2	NP	
19, M	5F-PB-22: 1.5 (*)	–		NP	Bilateral pulmonary edema and congestion of viscera	Necrotizing granulomatous inflammation with histoplasma microorganisms	Acute drug intoxication using the synthetic cannabinoid 5F-PB-22	NP	TSS 2	NP	
36, M	JWH-018: 0.1 JWH-122: 0.39 AM-2201: 1.4 MAM-2201: 1.5 UR-144: 6 (estimated)	Amphetamine: 250	Sudden collapse after smoking herbal blend named “Mary Joy Annihilation”	Seizures, multiple attempts of resuscitation	Stenosing coronary sclerosis, pulmonary and cerebral edema and congestion of inner organs	NP	Acute influence of several SCRAs and amphetamine	NP	TSS 1	Contributory	Schaefer et al. (46)
17, M	JWH-210: 12	–	Found dead outdoors (temperature during the night was 6–8°C), after having smoked a mixture of herbs labeled “Smoke XXX. A potent potpourri”	NP	Low BMI (16.4), lung edema	NP	Hypothermia in combination with SCRA intoxication	Hypothermia	TSS 2	Significant role	Kronstrand et al. (52)
23, M	AM-2201: 12, AM-2201 m: 2.47 JWH-018 m: 123, 50.8 (*)	–	A sibling heard 30 minutes of “stomping noises.” Significant damage to a wall and glass window at the scene, a large volume of blood covered the floor, windows and walls	No known history of mental illness, seizures disorder, previous psychiatric care or past or current use of illicit drugs or over-the-counter medication (OTC)	Multiple blunt-force injuries to both hands and sharp force wounds on the head and upper extremities, including a fatal stab wound at the neck	NP	Self-inflicted fatal wound due to SC use. No evidence indicating the intent of self-harm	Psychiatric complications caused by AM-2201 use	TSS 2i	Psychiatric complications caused by SCRA	Patton et al. (41)
26, M	AM-694: 0.09 (0.00009 µg/g) AM-2201: 0.3 (0.0003 µg/g) JWH-018: 0.05 (0.00005 µg/g)	Methoxetamine: 8600 Venlafaxine 300 O-desmethylvenlafaxine: 400, THC: pos	Found on the floor in his apartment	History of drug abuse and depression (treated with venlafaxine)	Pulmonary edema	NP	Acute fatal intoxication with methoxetamine		TSS 1	Possible contributory	Wikström et al. (42)
59, M	MAM-2201: 12.4 (^*^)	–	Found dead on a sofa at home	NP	Unremarkable	Unremarkable	Acute intoxication	NP	TSS 3	Fatal intoxication	Saito et al. (44)
57, M	JWH-018: 199 (*)	Clonazepam: 5.5 7-aminoclonazepam: 56.6 Methadone: 887 EDDP: 115 Morphine: 122 Pregabalin: 1800 Topiramate: 4100 Naloxone: pos (prescribed)	The man had smoked herbal incense (Spice) and a white powder presumably containing JWH-018	Unresponsive to Narcan, asystole	Enlarged heart	NP	NP	NP	TSS U	NP	Shanks et al. (43)
52, M	JWH-018: 19.6 JWH-073: 68.3 (*)	–	Avid herbal incense user, found nude and unresponsive at home	NP	NP	NP	NP	NP	TSS U	NP	
29, M	JWH-018: 83.3 (*)	–	Avid SCRAs user, history of suicidal tendencies	NP	NP	NP	Suicide by exsanguination	NP	TSS 3i	NP	

Ref, reference; age, -s: decade (e.g., 30s: from 30 to 39 years old); PBC, peripheral blood concentration; m, metabolites; pos, positive; NP, not provided; TSS, Toxicological Significance score.

^*^Quantified in central blood.

^**^Unclear if central or peripheral concentration.

-: no other compounds detected.

TSS indexed with “i” marks an indirect mechanism.

**Table 3:** Post-mortem concentrations in peripheral blood (PBC), central blood (CBC) and other tissues.

Cannabinoid(s)	PBC (ng/mL)	CBC (ng/ml)	PMI	Urine	Other organs concentration (ng/g)	Author, Year
EAM-2201, AB-PINACA, AB-FUBINACA	EAM-2201: 0.0566 ± 0.0042; AB-PINACA: 0.0126 ± 0.0001	EAM-2201: right heart 0.0287 ± 0.0045; EAM-2201: left heart 0.031 ± 0.0056; AB-PINACA: right heart 0.0196 ± 0.0038; AB-PINACA: left heart 0.0206 ± 0.001	2 days		EAM-2201: lung 0.35, liver 0.13; kidney 0.12; AB-PINACA: lung 0.36; liver 0.17; kidney 0.14; AB-FUBINACA: lung 0.12, liver 0.05, kidney 0.02	Yamagishi et al. (19) Minakata et al. (25)
5F-PB-22, 5F-AKB-48	.unquantified	unquantified	8 h			Langford and Bolton (20)
AB-CHMINACA	(subclavian) 8.2					Paul et al. (21)
UR-144, XLR-11, JWH-022	.UR-144: (subclavian) 12.3; XLR-11: (subclavian) 1.3; JWH-022: (subclavian) 3					Paul et al. (21)
5F-ADB, 5F-ADB-PINACA, MAB-CHMINACA	MAB-CHMINACA 6.05	MAB-CHMINACA: right heart 10.6; left heart 9.30	35-40 h	m		Hasegawa et al. (22, 29)
5F-ADB		0.19	2 days	m		Kusano et al. (23)
MDMB-CHMICA	1.7	2.1	12 h	0.01 + m	brain: 5.5; lung: 2.6; liver: 2.6; stomach content: 2.4; kidney: 3.8; psoas muscle: 1.2	Gaunitz et al. (24)
5F-ADB	(iliac) 0.12	right heart 0.24, left heart 0.45				Usui et al. (50)
5F-ADB	(iliac) 0.23	right heart 1.35				
5F-ADB	(iliac) 0.16	right heart 0.14, left heart 0.11				
5F-ADB	(iliac) 1.38	right heart 1.92				
AB-CHMINACA, 5F-AMB			2 days	AB-CHMINACA, 5F-AMB	AB-CHMINACA: brain 15.6, heart 20, lung 8.02, liver 21.2, spleen 7.55, kidneys 24.7, pancreas 38.9, adipose tissue 24.8; 5F-ADB: adipose tissue 18.7	Minakata et al. (25), Hasegawa et al. (28)
5F-ADB, MAB-CHMINACA			1 day	5F-ADB, MAB- CHMINACA		Minakata et al. (25)
UR-144	2.1					Rojek et al. (26)
UR-144	1.4					
UR-144	4					
5F-PB-22	0.37			m		Angerer et al. (27)
AB-CHMINACA	4.1			m		
5F-ADB	0.38					
AB-PINACA	26					Kubo et al. (47)
5F-AB-PINACA, 5F-AMB	5F-AMB: 8			5F-AMB: 176; 5F-AB-PINACA: 152		
Mepirapim	157			5200		
5F-AB-PINACA, 5F-AMB	5F-AMB: <1			5F-AMB: 28; 5F-AB-PINACA: 21		
AB-CHMINACA	1.5**		3 days	0.1	brain blood 2.2, lung blood 2.7, liver blood 0.3, kidney blood 1.3, intestines blood 1.0	Gieron et al. (31)
5F-AMB	(subclavian) 0.3					Shanks and Behonick (32)
JWH-018, AM-2201	JWH-018: 0.11; AM-2201: 2.5					Labay et al. (33)
JWH-210						
JWH-018, AM-2201, JWH-122, JWH-210	JWH-018: 0.65; AM-2201: 0.21; JWH-122: pos; JWH-210: pos					
XLR-11	pos					
JWH-018, AM-2201	JWH-018: 0.47; AM-2201: 17					
XLR-11, UR-144	pos					
JWH-018	pos					
JIWH-122, JWH-210, AM-2201	JWH-122: pos; JWH-210: pos; AM-2201: 0.16					
UR-144	pos					
JWH-210, AM-2201	JWH-210: pos; AM-220: 1.1					
XLR-11	pos					
XLR-11	pos					
XLR-11	pos					
AM-2201, JWH-018	AM-2201: 2.8; JWH-018: 0.11					
JWH-122, JWH-250, AM-2201	JWH-122: pos; JWH-250: 0.23; AM-2201: 7.3					
JWH-122	pos					
JWH-122, JWH-210, AM-2201	JWH-122: pos; JWH-210: pos; AM-2201: 0.22					
AM-2201	0.13					
JWH-122	pos					
XLR-11	pos					
XLR-11	pos					
JWH-175	105					
JWH-210	pos					
ADB-FUBINACA		inferior vena cava 7.3				Shanks et al. (51)
MDMB-CHMICA	1**					Seywright et al. (49)
MDMB-CHMICA	<1**					
Mepirapim	950 (serum, 3.5 h after use)					Fujita et al. (35)
MDMB-CHMICA	5.6 (ante-mortem), <0.2 post-mortem				brain 2.6, stomach content 0.2, bile < 0.2, kidney 0.2	Adamowicz (36)
MDMB-CHMICA	(ante-mortem serum) 1.4				spleen 0.1	Westin et al. (48)
AB-CHMINACA, AB-FUBINACA, AM-2201, 5F-AMB, 5F-APINACA, EAM-2201, JWH-018, JWH-122, MAM-2201, STS135, THJ2201, UR-144, XLR-11	AB-CHIMINACA: 2.8; AB-FUBINACA: 0.97; 5F-AMB: 0.19; 5F-APINACA: 0.51; STS 135: 0.16; THJ 2201: 0.16	AB-CHIMINACA: 1.1		m		Hess et al. (37)
MAM-2201, AM-1220, AM-2232	MAM-2201: 16.3; AM-1220: 140; AM-2232: 0.86 + m	MAM-2201: right ventricle 30.7, left ventricle 85.8; AM-1220: right ventricle 222, left ventricle 438; AM-2232: right ventricle, 1.76 left ventricle 1.95	20 h	AM-1120: traces		Zaitsu et al. (38)
XLR-11	1.4					Shanks et al. (34)
XLR-11	0.6					
PB-22						Gerostamoulos et al. (39)
PB-22						
PB-22						
5F-PB-22	1.1					Behonick et al. (40)
5F-PB-22	(ante-mortem) 1.3					
5F-PB-22	(iliac) 1.5					
5F-PB-22		superior vena cava 1.5				
NNEI	0.84-0.99	right atrium 0.75, left atrium 0.64	3 days		brain 0.76, heart 0.82, lung 1.06, liver 1.31, kidney 0.92, adipose tissue 42.9	Sasaki et al. (45)
JWH-210	12					Kronstrand et al. (52)
AM-2201, JWH-018		AM-2201: 12; AM-2201 m: 2.47; JWH-018 m: 123 and 50.8				Patton et al. (41)
MAM-2201		12.4	4 days		brain 4.3, liver 18.1, kidneys 11.2, adipose tissue 1535	Saito et al. (44)
JWH-122, JWH-018, JWH-210, MAM-2201, AM-2201, UR-144	JWH-018: 0.1; JWH-122: 0.39; AM-2201: 1.4; MAM-2201: 1.5; UR-144: 6 (estimated)					Schaefer et al. (46)
AM-694, AM-2201, JWH-018	AM-694: 0.09 (0.00009 µg/g), AM-2201: 0.3 (0.0003 µg/g), JWH-018: 0.05 (0.00005 µg/g)					Wikström et al. (42)
JWH-018, JWH-073		JWH-018: 199				Shanks et al. (43)
JWH-018, JWH-073		JWH-018: 19.6; JWH-073: 68.3				
JWH-018, JWH-073		JWH-018: 83.3				

PBC, peripheral blood concentration; CBC, central blood concentration; PMI, post-mortem interval; h, hours; m, metabolites; pos, positive, not quantified.

### Analytical Issues

Sample preparation and extraction procedures varied widely: liquid-liquid extraction was the most frequently used, though solid-phase extraction (24, 44, 47, 53) and QuEChERS dispersive solid-phase extraction (29, 30, 35, 50) were also reported. Only in a minority of the cases, the standard addition method was employed for quantification (19, 23, 28, 30). In two cases, liquid chromatography quadrupole time-of-flight mass spectrometry (LC-QToF-MS) was used to detected and quantitate parent compounds and/or metabolites in blood and ante-mortem serum (38, 48).

Overall, 31 SCRAs (EAM-2201, AB-PINACA, 5F-PB-22, 5F-AKB-48, 5F-ADB, AB-CHMINACA, UR-144, XLR-11, JWH-022, MAB-CHMINACA, MDMB-CHMICA, 5F-AMB, Mepirapim, JWH-018, AM-2201, JWH-210, JWH-122, JWH-250, JWH-175, ADB-FUBINACA, AB-FUBINACA, 5F-APINACA, MAM-2201, STS135, THJ 2201, AM-1220, AM-2232, PB-22, NNEI, AM-604, and JWH-073) were detected, some being more frequently identified in the revised cases, such as 5F-ADB, XLR-11, AM-2201, AB-CHMINACA, and JWH-018.

Even if reported with lower rates, 5F-PB-22, UR-144 were also common. XLR-11 was mostly reported in 2016, while 5F-ADB showed a peak in 2017 and 2018. However, a clear trend cannot be determined on the sole basis of this data.

While some laboratories applied national or international validation guidelines, such as those of the German Society of Toxicological and Forensic Chemistry (GTFCh), “in house” methods have also been adopted, stating overall good results (31, 32, 43). Analytical details were not always given and not all of the above-mentioned parameters, especially matrix-effects, were systematically assessed (19, 27, 31, 37, 38, 41, 44, 52).

According to previously published cases (33, 43), concentrations of SCRAs in post-mortem cases covered a wide range, from 0.01 (19) to 199 ng/mL (43), although lower concentrations, in the range 0.5 to 2.5 ng/ml, were most frequently encountered.

Peripheral blood was analyzed in 53 cases out of 74 (72%). In 8 cases (11%) only heart blood concentrations were stated, while both peripheral and central concentrations were published in 10 cases (14%). Other biological matrices, apart from urine, were quantitatively analyzed in only 8 cases (11%) (**Table 3**). Other substances were found in 44 out of 74 cases (59%).

As regard other xenobiotics detected, ethanol was detected in 13 cases (17%), though levels ≥ 1.5 g/L were found only in 6. A co-consumption of NPS, as synthetic cathinones (pentedrone, α-PVP, DL-4662), hallucinogens (6-APB, 6-MAPB, methoxetamine), anesthetics (diphenidine), and synthetic opioids (AH-7921) was seen in 11 cases (15%). Common drugs of abuse detected included antidepressive/neuroleptics/antipsychotics (quetiapine, trimipramine, olanzapine, sodium valproate, mirtazapine, amitriptyline, phenytoin, paroxetine, aripiprazole, citalopram, fluoxetine, haloperidol, trazodone, venlafaxine, pregabalin, topiramate) (12/74, 16.2%), cannabinoids (10/74, 13%), amphetamines, benzodiazepines (both 6/74, 8%) and opioids (5/74, 7%).

### Case Reports

**Figure 2:**
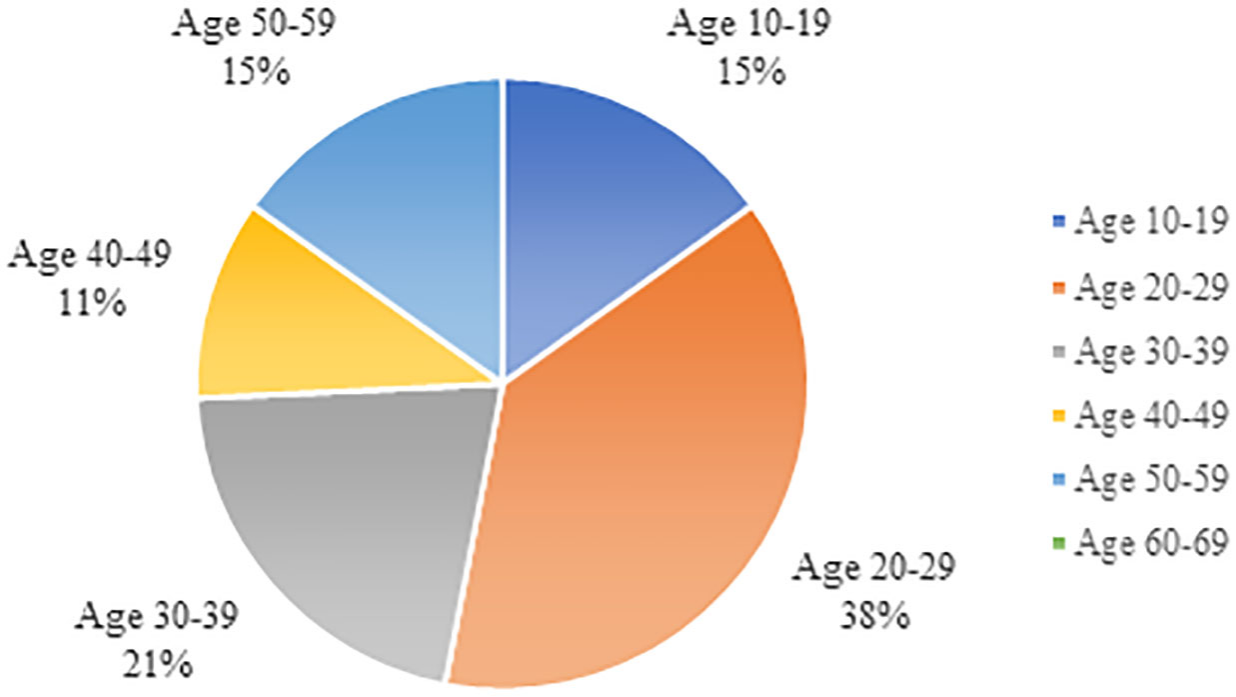
Age distribution among the cases reported in the literature.

The age of the deceased ranged from 14 to 61 (19). Mean age was 32, median 29. The 38.5% belonged to the 20 to 29 decades. Teenagers were also represented (15.4%) (**Figure 2**). With reference to the gender of the victims, 88.1% were male and 11.9% were female. A past use of drugs and/or alcohol was reported in 18 out of 74 cases (24%), while poor mental health was only reported in 5 cases (7%).

Herbal blends, smoking devices (e.g., pipes) and other paraphernalia were found during the death scene investigation (DSI) in 30 of 74 cases (41%) and were variably labeled as “Aladdin platinum/limited,” “Herbal incense, the super lemon,” “F1,” “Hammer Head,” “Magic Gold,” “Desert Premium Potpourri,” “AL 37,” “AP 31,” “Strongman,” “GM sapphire,” “Heart Shot Black,” “Apollo,” “Mocarz,” “Smoke XXX. A potent potpourri,” “Mad Hatter Incense,” “Fairy evolution,” “Mary Joy Annihilation,” “Passion Flower Herb – Zonk,” “Stoner Pot-Pourri K11,” “Supanova Pot-Pourri,” “K2 Cherry,” “Space Cade Flight Risk,” “Game over,” “Orange Flame,” “Legal Phunk,” “Mojo.” Clinical data was available in 15 cases (20%) and mostly included the detection of cardiac arrest/asystolia/fibrillation at the arrival to an Emergency Department.

Results of post-mortem examination, as for macroscopical or gross findings, were available in 55 cases (74%), including those cases in which only a short referral to “unremarkable findings” was reported. In most cases in which a SC was later discovered during toxicological analysis, the post-mortem examination had revealed only non-specific signs of intoxication, such as pulmonary edema and congestion, brain edema, hemolysis, and signs of aspiration (34, 40). In some cases, stomach and gastroduodenal erosions (20, 35), abundant hypostasis coupled to petechiae (27, 29, 37) and intracutaneous skin bleedings (vibices) (30) were reported. Lastly, cardiac abnormalities, such as cardiomegaly (33, 43), dilatative or hypertrophic cardiomyopathy (21), stenosis due to atherosclerosis (33, 46) or acute thrombosis of the coronary arteries were seen (32).

Histological data, as a specific result of a microscopical analysis, was clearly described only in 13 cases (18%).

Cause of death was stated in all but 4 cases (5%). Cardiac arrhythmias and cardio-circulatory acute effects were listed in 17 cases (23%) as cause of death or as underlying mechanisms in a group of mono and poly-drug intoxications, involving both teenagers and adults (20, 21, 35, 40, 48). A number of death cases were associated with excited delirium and police restraints (33), as well as fall from height, either due to drug-induced psychosis or reduced awareness with accidental falling (24, 26). Behavioral effects could also lead to suicide (26), self-inflicted self-injuries (41) and further consumption of other drugs (26, 55). Respiratory depression (27), especially in the setting of mixed intake of xenobiotics (33, 51), and asphyxia due to aspiration of gastric content in a state of coma (22, 27, 28) were also declared.

Manner of death was mostly accidental or not given and 4 cases of suicide (5%) were recognized.

Post-mortem interval (PMI) was stated in 11 cases (15%) and ranged from 8 hours to 4 days (**Table 3**).

## Discussion

The systematic review of the literature has resulted in an unexpectedly high number of cases of death involving SCRAs. Though, given the widespread use of the compounds, similar fatalities might be under-reported (publication bias) or under-recognized as a result of the challenges of post-mortem analyses. Indeed, the delay in developing and updating analytical methodologies affects the capability of many laboratories to detect and report cases of SCRA-related death, particularly when novel compounds which just entered the market are involved (55). Accordingly, the results here presented cannot be taken to estimate the prevalence.

### Circumstantial Data

In almost the totality of cases, a possible involvement of SCRAs was suggested by either a past history of drug/alcohol abuse, by witnesses' statements of partying or smoking shortly before collapse, or by the DSI, which revealed paraphernalia and herbal residues. Depending on the market availability, it emerged that the content of such packages varied over time (31, 36, 55). Thus, it must be kept in mind that the product names do not validly predict the ingested substances.

Although e-cigarettes and e-liquids represent an innovative and attractive way to consume SCRAs (56, 57), no vaping liquids were reported in the circumstantial data of the reviewed death cases. The link between e-cigarettes and SCRA consumption might be less familiar and known to the investigators, possibly resulting in such liquids not being systematically seized and/or analyzed. When such paraphernalia are found, an analysis of vaping liquids collected at the DSI should be strongly encouraged.

SCRAs are not detected by common immunoassays and require a target analysis, which is usually only requested by authorities and conducted when a suspicion is raised, due to analytical limitations and economic reasons (21). This factor could lead to a failure to recognize such cases and consequently to a massive underestimation of the number of death cases involving SCRAs. This underlines the importance of an appropriate awareness and of an in-depth experience in forensic toxicology during DSI and when interviewing witnesses.

### Analytical Issues

The data extracted from the revision of the literature regarding toxicological analyses once again highlights the role of liquid chromatography-mass spectrometry (LC-MS/MS) for the quantification of SCRAs in biological specimens, although a singular preferred method for sample extraction is not known, given the variety of chemical differences among analytes (52). The use of standard addition methods, as recently suggested in a series of intoxications (58) is limited, possibly due to the low amount of post-mortem blood collected at the autopsy.

Only a small number of SCRAs emerged from the literature review, compared to the quantity of compounds included in the NPS category. We do not believe that this is a bias due to an attention to recent molecules, since the follow-up period ended in 2019 and more recent molecules, such as SCRAS bearing a γ-carbolinone core, were not found. This observation could be on the contrary related to difficulties in detecting SCRAs in the absence of a dedicated and updated method. The main challenge in forensic toxicology from an analytical point of view resides indeed in keeping methods updated, in order to detect novelties as soon as they are involved in death cases. Although there was a decrease in the last years, the frequent emergence of novel compounds might lead to missing relevant analytes. This was clearly demonstrated by cases where re-analysis of samples with novel and more sensitive methods allowed for identification of substances previously undetected (19, 22, 25). Very sensitive methods are needed in blood, given the low concentrations reported in the literature (52) and urine analysis can reveal a previous intake even when nothing is detected in blood (22, 23).

Once a new methodology for the analysis of biological specimens has been established, even in case reports, a so-called short validation, including selectivity, linearity, accuracy, precision, and matrix effect, is strongly recommended even if not always performed or published (59). In the case described by Langford et al., for example, the analyses were granted by a private licensed forensic laboratory, and no information was disclosed due to alleged “competition and market issues” (20). The absence of a clearly stated methodology and validation process questions the reliability of the analytical results and limits the comparability of the data. The validation could be further hampered by the lack of material for re-analysis and/or by the lack of isotopically labeled standards (27, 33). Lack of material represents a serious limitation particularly in the case of measured concentrations being far above the linear range of the calibration, even though results might be estimated by extrapolation (27, 38, 43). It has to be underlined that the vast majority of the methods for SCRAs quantification were validated in serum and not in post-mortem blood.

When evaluating the concentrations, several aspects need to be considered, including site of sampling, post-mortem interval, possible tolerance of the user, co-consumption of other drugs, potency of the compounds, chemical characteristics, and time delay between intake and death.

Some compounds, such as 5F-ADB, are known to be particularly unstable (29) and this could explain the extremely low concentrations of the highly potent SCRAs in our review (27). Rapid degradation due to pyrolysis, ante-mortem drug metabolism, as well as post-mortem redistribution and degradation (due to remaining esterase activity) were considered as further main factors for reduced concentrations and should be considered for all SCRAs, though with different weighting (19, 23, 44).

When compared to others SCRAs, mean concentrations were relatively higher for AB-CHMINACA, despite its high potency (21, 27). This could be due to the narrow time interval between drug smoking and death, approximately an hour in the case described by Paul et al. (21), or to a high tolerance of the subject implying high doses (21).

Concerning post-mortem redistribution (PMR), disparities among central and peripheral blood levels were mostly slight (19, 45) (PMI: 2 days). A 1.2 quotient of central/peripheral blood (C/P ratio) was found in a case of MDMB-CHMICA 12 h after death, and this result was interpreted as not indicative of PMR (24). Similarly, concentrations were in the same range in all tissues in a case described by Yamagishi et al. (19). On the contrary, cardiac blood levels strongly exceeded the peripheral ones in the case of Zaitsu et al. (38) for MAM-2201, AM-1220, and AM-2232 (PMI: 20 h), where left and right ventricle blood levels were 2 to 5 and 1.5 to 2 times higher than femoral blood concentrations. Quantification employed LC-QTOF, a full validation was not performed and concentrations of some analytes were far above the highest calibration point. Nevertheless, C/P ratios appear to tend to values above 1 especially in the case of short time intervals between smoking and death (in this case 1.5 h). A death shortly after smoking, with high concentrations of SCRAs in lungs being released to the surrounding vessels and tissues could explain the higher levels in the blood of the left ventricle (60), although the authors suggested a myocardial accumulation instead (38). C/P ratios of 1.75 to 1.54 were also seen by Hasegawa et al. (29) (PMI: 2 days).

Divergences among compounds suggest that the chemical characteristics (e.g., greater or lower lipophilicity) as well as the pattern of use should be considered when hypothesizing PMR (45). Ingestion and smoking probably result in higher concentrations in stomach content and lungs, respectively. This could lead to a release into nearby vessels of the central compartment. Since femoral blood levels increase mostly due to release/redistribution from fat and muscle tissues, an inversed central/peripheral ratio would suggest a greater lipophilicity of the compound and/or chronic accumulation of SCRAs in deep compartments. However, the scarce information regarding time between last consumption and death as well as the PMI complicates the situation. Stability and matrix effects additionally limit the capability of drawing valid conclusions based on blood concentrations.

The distribution of SCRAs in tissues varied widely. Concentrations in tissues have been assessed only in a minority of cases and results may strongly depend on the employed methods of analysis, the description of which is beyond the scope of this article. Given the rarity of this type of measurement, which afford a time-demanding standard addition method (19), the available data regarding tissue distribution does not allow for general conclusions. However, they can be used to evaluate the specific case and might allow identifying potential sites of accumulation. For example, extremely high levels in the adipose tissue were seen for MAM-2201 (124 fold higher than blood concentration), leading to the suggestion of fatty tissue as a suitable target specimen for analysis (PMI: 4 days) (44). High adipose levels were also found for NNEI (45) (PMI 3 days) and might be interpreted as a result of continued substance use. According to Kusano et al. (23) adipose tissues are a suitable matrix for the detection of the parent compound, while other tissues (with higher esterase activity) could contain higher levels of metabolites.

High levels in liver and kidney tissue could be found in the case of more hydrophilic compounds (e.g., MAB-CHMINACA; PMI: 2–3 days) (29), especially when the interval between intake and death was short. In these cases, an accumulation in fatty tissue might not have occurred yet, requiring longer time (24, 29). Brain concentrations were high for MDMB-CHMICA in a case described by Gaunitz et al. (PMI: 12 h) and this allowed to confirm that the victim was under the effect of cannabinoids at the moment of death (24). High concentrations in lungs were also reported, pointing towards an intake through smoking.

In summary, blood and tissue concentrations should always be interpreted with caution, due to the multiple factors which have to be taken into account (e.g., PMR, PMI, active metabolites, stability, chemical characteristics, plasma/blood ratio, tolerance etc.) (61).

### Pathology and Histopathology

The un-specificity of gross pathological findings heightens the risk of missing deaths involving SCRAs. Vomiting and aspiration of gastric content are highly suggestive for drug-induced coma or loss of consciousness in otherwise healthy and young subjects (22, 29). This was also seen in a case in which, originally, only urine was found positive for SCRAs, and further analyses demonstrated high blood concentrations of MAB-CHMINACA (22, 29). It should always be kept in mind that acute gastrointestinal bleedings could be due to several diseases and factors, such as Mallory-Weiss disease or hypothermia, and an accurate differential diagnosis remains fundamental before attributing such findings directly to SCRA consumption. For example, erosions seen in the case described by Langford and Bolton (20), could have been the result of ethanol intake, which was in his fatal ranges (3.11 g/L).

Bleedings and abundant hypostasis could raise the suspicion for a recent intake of SCRAs. In 2018, fatal and life-threatening bleedings were connected to superwarfarin-type drugs such as brodifacoum added to products allegedly containing SCRAs as reported by the Centers for Disease Control and Prevention (CDC), which released a health advisory. Long-acting anticoagulant rodenticides (LAARs) were occasionally found as adulterants in herbal blends (62). A case of death connected to anticoagulants is described in the literature (63), even though the case was not included in the review, since the past use of SCRAs emerged only from circumstantial data (thus, the paper did not fit into the inclusion criteria). It is not clear if hemolysis, abundant hypostasis, and intracutaneous or soft tissue bleedings can be caused by such adulterants or represent a hematological effect, maybe liver-mediated, of SCRAs themselves (40). As LAARs are usually not detected by urine screening analyses, highly sensitive LC-MS/MS methods are required for their detection (62).

The interpretation of cardio-vascular findings is discussed in the following subsection.

### Cause and Mechanism of Death, TSS

Several preclinical studies and case reports addressed the increased cardiovascular risk related to SCRAs use, though scientific evidence is still limited (14, 64–68). This was reflected by our literature review, since abnormal findings in heart were seen and death related to acute cardiovascular arrest or collapse certified. However, it could be unclear if these deaths are actually related to SCRAs or not (69). Marijuana exerts some cardiovascular effects, acutely resulting in increased catecholamine release and, consequently, increased heart rate and vasodilation, with orthostatic hypotension (68). Cannabis is told either to exert negligible effects on blood pressure or an increase in blood pressure, and might increase the risk of myocardial infarction, particularly in predisposed subjects (68, 70–72). SCRAs, being more potent cannabinoid receptor agonists, have been linked to the occurring of myocardial infarction (64) even in the absence of coronary artery disease, and of arrhythmia-related sudden cardiac death (65–67). Sasaki et al. (45) found signs of hypertension and aging in a 20-year-old victim, coupled to myocardial suffering, and thus hypothesized a cardio-circulatory hyperactivity due to prolonged SCRA use. The diversity of potential injury mechanisms (between myocardial infarction and arrhythmia) may explain why in some cases band necrosis proved a myocardial damage (21, 45), while in others neither macroscopic nor microscopic signs were noted (20, 21, 35).

In the cases where post-mortem examination failed to identify signs of heart diseases, the attribution of cardiac death to the SCRA was mostly based on circumstantial data, e.g., the victim reported having smoked shortly before dying and/or a sudden collapse after smoking occurred (20, 35, 40). In a similar case, the possible role of SCRAs was confirmed by a serum sample collected only 2 h after a sudden collapse with asystolia, revealing 1.4 ng/mL of MDMB-CHMICA (48).

Moreover, asystole was initially noted in a case of death where, after resuscitation, a multi-organ failure finally led to cardiac arrest (36).

Atherosclerotic disease and other cardiac abnormalities, such as cardiomegaly and dilatative cardiomyopathy, pose an additional challenge to the assessment of the role of SCRAs in death cases (50, 61). In fact, drugs can either exacerbate a pre-existing condition, or be considered an irrelevant finding (33). In a case described by Tse et al. (64) a triple-vessel coronary thrombosis was found and death was attributed to myocardial infarction with a possible contributory role of SCRAs, despite the absence of analytical confirmation. In a case involving ADB-FUBINACA, Shanks et al. (51) concluded that, due to occurrence of behavioral effects followed by a sudden death, a SCRA-induced dysrhythmia contributed to the death, notwithstanding the presence of a potentially fatal thrombolytic occlusion. Similarly, a contributory role was stated by Tse et al. (64) despite morphological findings which could have explained the death by themselves. These cases demonstrate that it can be difficult to assess the cardiac effects of SCRAs, particularly in the presence of findings potentially constituting a cause of death on their own. In such cases, a TSS of “1,” which does not exclude a partial contribution, seems to be appropriate (17).

In cases of polydrug abuse, the evaluation of role cannot leave aside concentrations of xenobiotics, leading to attribution of different TSS on a case-by-case basis, e.g., TSS U with 3 g/L of ethanol, positive unquantified SCRAs and suspected arrhythmias (20) vs TSS 3 with 1.8 g/L ethanol, 1.5 ng/mL AB-CHMINACA and acute cardiorespiratory depression (50).

Coma/somnolence can lead to death directly, through vomiting/aspiration or indirectly due to environmental exposure and hypothermia (54). In a case presented by Kronstrand et al. (52), death occurred due to “hypothermia and SCRA use,” despite the absence of typical hypothermia-related signs such as freeze-erythema and Wischnewsky spots (52). Hypothermia after the use of SCRAs was seen in experimental studies on animals (e.g., male rats and monkeys) (73, 74) and has been partially related to the effects of cannabinoids on dopamine receptors (75), but has not been confirmed in humans yet. However, in a case described by Adamowicz (36) a cadaveric temperature of 35.1°C was measured, despite the victim was at home and the measurement took place only 30 minutes after the sudden collapse. This finding would be in line with the animal experimental data regarding the effect of SCRAs on body temperature and highlights the need to evaluate body and ambient temperatures in cases of death possibly related to such compounds. An “intoxicated-state” was also considered as the underlying mechanism of a death due to ketoacidosis, even though an AB-CHMINACA-induced hyperglycemia was also possible (37).

A behavioral contribution of SCRAs to the death appears to be an additional source of concern. Anxiety and psychosis might be also explained by the affinity of SCRAs to dopaminergic (D2), serotoninergic (5-HT2A) or glutamatergic (NMDA) receptors (76, 77). In a case described by Labay et al. (33), the victim fell from a high building after feeling sick and vomiting several times and was found intoxicated with MDEA, MDA, and JWH-175. While psychiatric consequences of SCRAs intake are clear in the absence of other drugs (41), the evaluation of role in polydrug consumption is puzzling, as in the case of low levels of both SCRAs and phenytoin, which can induce psychosis (78). Data on toxic/fatal levels might lack for NPS (e.g., for co-consumption of pentedrone resulting in behavioral abnormalities) (26) and even therapeutic or negligible levels of common drugs of abuse could assume relevance in combination with SCRAs. The influence of SCRAs on non-cannabinoid receptors and on serotonin, dopamine, catecholamine levels further complicate potential interpretations. In such cases of polydrug abuse, it is possible that the death would not have occurred without SCRA consumption, although no direct causality can be established. A TSS of “1” (“i” indicating the indirect role) is suggested due to the presence of multiple drugs with unknown contributory role, despite behavioral toxicity being an important risk factor for fatal outcome (33).

On the other hand, in the first case reported by Rojek et al. (26), the victim jumped from a building after a reported “loss of control” and no other drug was detected. Thus, notwithstanding the behavioral toxicity and the indirect mechanism, a contribution to death is likely (TSS of “3” was assigned).

Finally, cases of acute liver and/or kidney failure have been described (40, 55).

In general, if only toxicological results are listed in the absence of macroscopical and microscopical data, uncertainties regarding the role of the substance increase, as in the case reported by Kusano et al. (23). However, the mechanism of death could remain unclear, despite having a more complete data set (40) and the agreement between independent reviewers judging the very same pieces of information could be weak (e.g., unanimous agreement in 2 cases out of 25 submitted to multiple evaluations) (33, 40). Thus, a multidisciplinary evaluation should be recommended for each case, in order to possibly limit such uncertainties.

Most of the publications identified a possible contributory role of SCRAs, even in the absence of findings clearly pointing towards a drug-related death (32, 34, 39, 45, 48). In the present review, a TSS of 3 was assigned when no competitive cause was seen, and the hypothesized mechanism of death was in line with the most frequently reported SCRA toxicities. Affinity and activity of new compounds are often unknown, and unexpectedly severe or idiosyncratic effects may occur (19). Given the uncertainty regarding toxic levels and toxic effects, even when the mechanism of death remains unclear and/or other substances might have played role, the possibility of a contribution of SCRAs should not be ignored, and a TSS of “2” justified.

The likelihood of a high significance score is greater when multiple compounds with potentially synergic effects are detected, despite low concentrations of each single compound (19).

On the contrary, even though SCRAs could exacerbate an intoxication due to alcohol or other drugs, in cases with relatively very low SCRA concentrations or with concentrations of the competitive drug above the toxic threshold, a TSS of “1” is suggested. This does not necessarily mean that the SCRA has not exerted any negative effect, and the stability of the analyte of interest should further be considered as a cause of low concentrations.

TSS was rated “U” in cases with lack of sufficient data, as for example in the case described by Langford and Bolton (20), where high alcohol concentrations were retrieved, and no quantification of SCRAs was possible, or in the case of Minakata et al. (25) and Kusano et al. (23), where the effects of the other substance detected was difficult to assess.

## Limitations

There are several limitations in this study. First of all, despite the extensive research involving multiple databases, the process of inferring scientific evidence is strongly limited by the possibility of under-reporting of similar cases and by the necessity of establishing a temporal limit for the review. Thus, the information presented have to be regarded as incomplete. Secondly, no weighting of selected articles regarding their quality was undertaken. A third significant limitation resides in establishing the TSS (16) of the selected cases, since the TSS is so far a non-validated scale. However, given the lack of criteria for establishing the role of substance(s) in death cases, the TSS appeared to be a flexible and easy-to-use tool to assign a contributory weight to SCRAs, and thus to evaluate and compare different cases. In order to avoid misinterpretations and to appreciate the point of view of the authors of the manuscripts, in each death case the role of the substance was also supplied in their own words. Finally, only death cases in which at least one SCRA was analytically confirmed, and in which an autopsy was presumably performed, were included. The authors are aware that this could have resulted in a partial loss of information, but on the other hand our aim was to possibly achieve a higher level of evidence.

## Conclusions

Several mechanisms could lead to death after SCRAs consumption, and behavioral risks as well as cardiovascular effects or central nervous system depression appear to play important roles. Given the limited pharmacodynamic and pharmacokinetic data and the overlap between fatal and non-fatal concentrations, typical toxic ranges for SCRAs have not been identified so far. The results of toxicological analyses should be interpreted with caution, considering the many confounding and influencing factors, particularly regarding the reliability of LC-MS/MS methods validated insufficiently or validated only in serum. Furthermore, pattern of consumption (e.g., occasional v. chronic) and tolerance of the subject should be estimated or evaluated on a case by case basis.

A complete and accurate post-mortem examination is a fundamental part in the evaluation of death cases involving SCRAs, since a comprehensive and multi-disciplinary evaluation of clinical, circumstantial, toxicological, and autoptic data is the only possibility to assess the toxicological significance of a substance and to tentatively identify a plausible mechanism of death, which could remain unclear despite an in-depth analysis of all data available.

## Author Contributions

All authors materially participated in the article preparation and have approved the final article.

## Funding

Role of funding source: The study was supported by Presidency of the Ministers Council, Department of Antidrug Policy.

## Conflict of Interest

The authors declare that the research was conducted in the absence of any commercial or financial relationships that could be construed as a potential conflict of interest.
